# Structural insights into the substrate tunnel of *Saccharomyces cerevisiae *carbonic anhydrase Nce103

**DOI:** 10.1186/1472-6807-9-67

**Published:** 2009-10-24

**Authors:** Yan-Bin Teng, Yong-Liang Jiang, Yong-Xing He, Wei-Wei He, Fu-Ming Lian, Yuxing Chen, Cong-Zhao Zhou

**Affiliations:** 1 Hefei National Laboratory for Physical Sciences at Microscale and School of Life Sciences, University of Science and Technology of China, Hefei, Anhui, 230027, PR China

## Abstract

**Background:**

The carbonic anhydrases (CAs) are involved in inorganic carbon utilization. They have been classified into six evolutionary and structural families: α-, β-, γ-, δ-, ε-, ζ- CAs, with β-CAs present in higher plants, algae and prokaryotes. The yeast *Saccharomyces cerevisiae *encodes a single copy of β-CA Nce103/YNL036W.

**Results:**

We determined the crystal structure of Nce103 in complex with a substrate analog at 2.04 Å resolution. It assembles as a homodimer, with the active site located at the interface between two monomers. At the bottom of the substrate pocket, a zinc ion is coordinated by the three highly conserved residues Cys57, His112 and Cys115 in addition to a water molecule. Residues Asp59, Arg61, Gly111, Leu102, Val80, Phe75 and Phe97 form a tunnel to the bottom of the active site which is occupied by a molecule of the substrate analog acetate. Activity assays of full length and two truncated versions of Nce103 indicated that the N-terminal arm is indispensable.

**Conclusion:**

The quaternary structure of Nce103 resembles the typical plant type β-CAs of known structure, with an N-terminal arm indispensable for the enzymatic activity. Comparative structure analysis enables us to draw a possible tunnel for the substrate to access the active site which is located at the bottom of a funnel-shaped substrate pocket.

## Background

Carbonic anhydrase (CA; *EC 4.2.1.1*) is a zinc containing enzyme which catalyzes a reversible reaction to form bicarbonate from carbon dioxide and water [1,2]. By facilitating the interconversion between CO_2 _and bicarbonate in *vivo*, CA activity was proposed to be required to ensure adequate level of CO_2_or HCO_3_^- ^as the substrates for other enzymes [3]. Therefore, CAs have been found to play an essential role in a series of fundamental biological processes such as photosynthesis, respiration, pH homeostasis and ion transport [4]. Based on the origins and structural features, CAs are divided into six classes, termed α, β, γ, δ, ε and ζ, respectively [5]. The α-CAs were found in mammals, algae and prokaryotes to facilitate the exchange of CO_2 _in the respiratory cycle. The β-CAs were characterized in higher plants, algae and prokaryotes, and those in higher plants have been reported to play a critical role during photosynthesis [6]. The γ-class was first identified in the thermophilic archaeon *Methanosarcina thermophila *[7], whereas δ- [8] and ζ- [9] class were found in diatom only. Notably, the ε-CAs from bacteria can form a carboxysome [10].

The β-CAs of known structure form a dimer, tetramer or octamer of identical subunits, with the active site located at the interface of two subunits. Each subunit consists of three parts, an N-terminal arm composed of two or three projecting α-helices, a conserved zinc-binding core consisting of α-β-α units exhibiting a Rossmann fold and an anti-parallel β-sheet and a C-terminal domain that is dominated by α-helices [3,5,11-15]. The N-terminal arm of one subunit makes significant contacts with another subunit via domain swapping. The overall structure of the zinc-binding core is relatively conserved, but the zinc-coordinating residues differ in various classes of CA. In β-CA, the zinc ion binds to two cysteines and a histidine, in addition to a water molecule or an aspartate as the fourth ligand. Based on the divergence of the active site, β-CAs were further grouped into two subclasses, the plant type and the cab type [16].

The budding yeast *Saccharomyces cerevisiae *encodes a single copy of CA, termed Nce103 after the non-classical export pathway where it was identified for the first time [17]. Deletion of *NCE103 *leads to a growth-defect phenotype under aerobic conditions, but not under anaerobic conditions [18]. The Δ*NCE103 *strain exhibits an enhanced sensitivity to H_2_O_2 _compared to the wild type [19]. However, the restoration of Δ*NCE103 *is due to the recovery of carbonic anhydrase activity rather than antioxidant activity [18]. Nce103 is an efficient β-CA who shows high CO_2 _hydrase activity, with a kcat of 9.4 × 10^5 ^s^-1 ^and kcat/Km of 9.8 × 10^7 ^M^-1 ^s^-1^[20].

**Figure 1:**
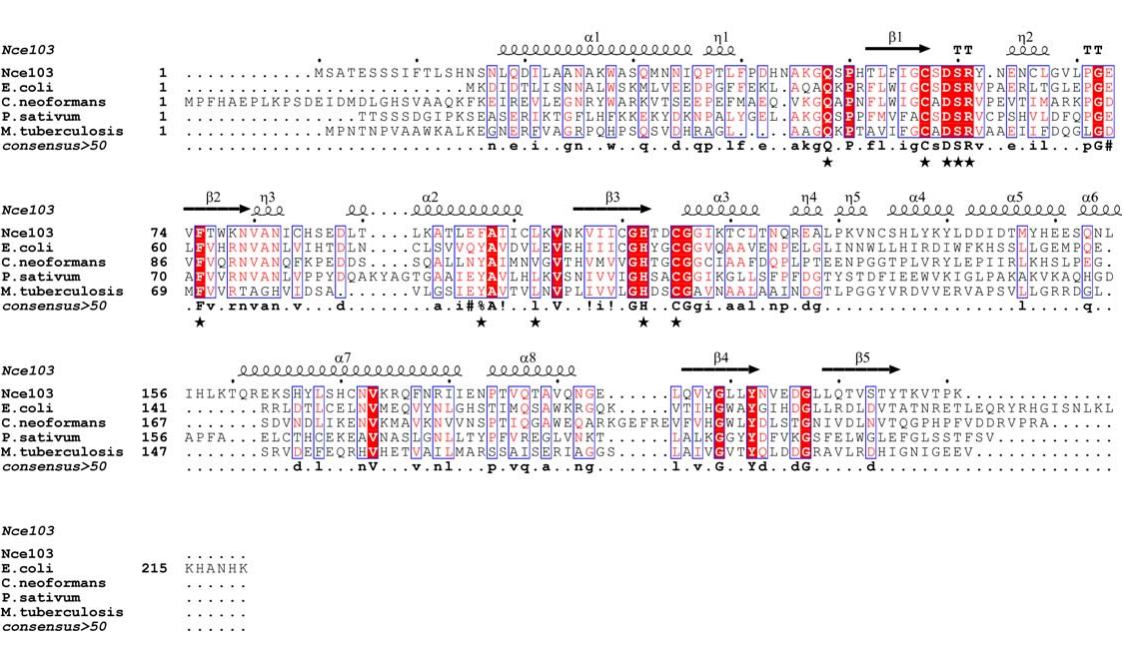
**Multialignment of Nce103 against carbonic anhydrases from *E. coli *(Swiss-Prot**: P61517**), *C. neoformans *(EMBL**: Q314V7**), P. sativum (Swiss-Prot**: P17067**), and M. tuberculosis (EMBL**: O53573**)**. Conserved residues at the active site are labeled with black stars at the bottom. All sequences were obtained from NCBI databases. Multialignment was performed using the programs MultAlin [25] and ESPript [26].

Nce103 contains all conserved residues of the active site in the plant type β-CAs, and a relatively divergent N-terminal region (Figure 1). To verify if this N-terminal region is critical for the CA activity, we overexpressed and purified three versions of Nce103, full length, Nce103ΔN13 and Nce103ΔN50. Moreover, we determined the crystal structure of Nce103ΔN13 at 2.04 Å resolution. Structural analysis in combination with comparative activity assays revealed the N-terminal region comprising two α-helices is indispensable for controlling the substrate tunnel.

## Results and discussion

### Overall structure

We overexpressed, purified and crystallized the full length Nce103, but failed optimizing the crystal to diffraction quality. After performing a multiple sequence-alignment, we deduced the first non-conserved 13 residues of Nce103 might interfere the crystal packing (Figure 1). Thus, we constructed the truncated version Nce103ΔN13 which enabled us to determine its crystal structure at 2.04 Å resolution.

**Figure 2:**
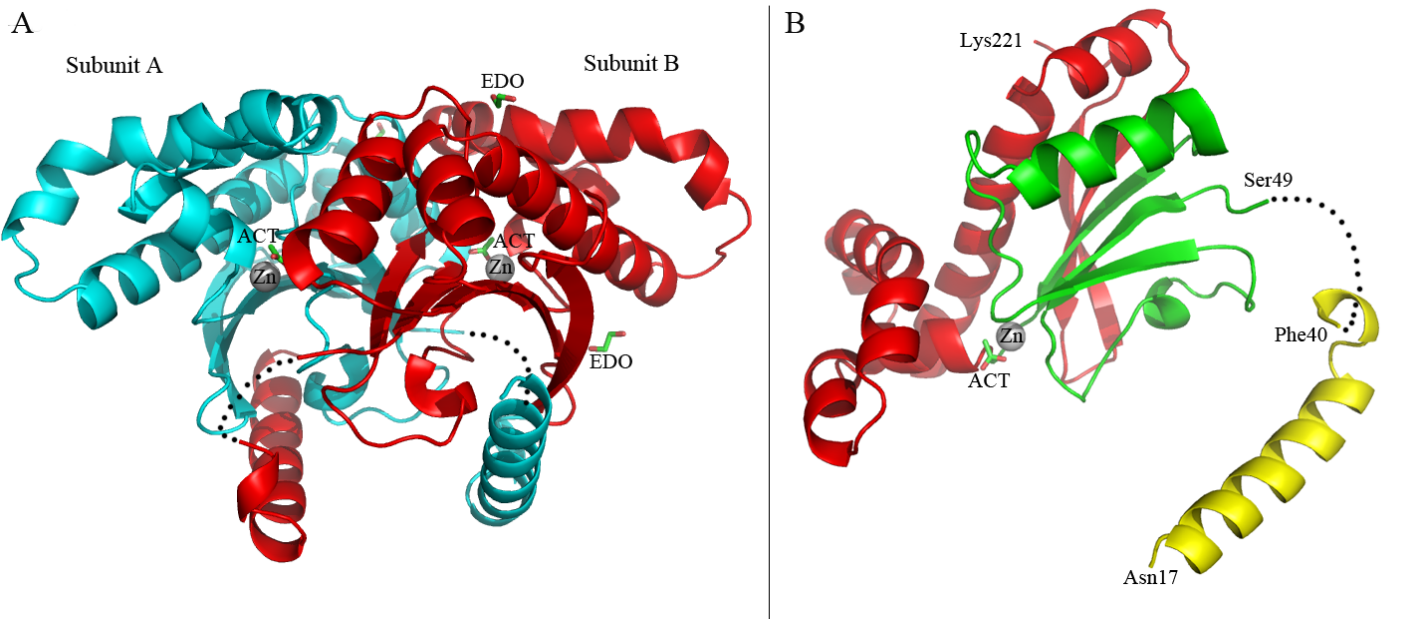
**The overall structure and organization of Nce103**. **A) **The cartoon representation of the Nce103 homodimer. The zinc molecules are shown as spheres and colored in gray. Subunits A and B are colored in cyan and red, respectively. **B) **The cartoon representation of the Nce103 monomer. The conserved α/β core and the C-terminal subdomain are colored in yellow, green and red, respectively.

The structure of Nce103ΔN13 resembles that of other plant type β-class CAs (*P. sativum*, PDB code: 1ekj[11]; *P. purpureum*, PDB code: 1ddz[12]; *E. coli*, PDB codes: 1i6p[3] and 1t75; *H. influenzae*, PDB code: 2a8c[5]; *M. tuberculosis *Rv3588c, PDB codes: 1ym3[13] and 2a5v[14], *C. neoformans*, PDB codes: 2w3n and 2w3q[15]). It comprises all the conserved active site residues (Gln48, Phe75, and Tyr/Phe97) as found in the plant type CAs. The overall structure of Nce103 contains two molecules in an asymmetric unit (Figure 2A). The missing electron density of the N-terminal 6× His-tag, residues His14-Ser16 and Pro41-Gln48 of each subunit indicates their high degree of flexibility (Figure 2B). Similar to other β-CAs, the Nce103 monomer consists of three components: an N-terminal arm, a conserved α/β core and a C-terminal subdomain (Figure 2B). The N-terminal arm is composed of two α-helices that extend away from the rest of the molecule, making extensive contacts with the other molecule in the asymmetric unit. The α/β core consists of a parallel four-strand β-sheet ordered β2-β1-β3-β4, with a fifth anti-parallel strand β5 connected by a short turn. The C-terminal subdomain contains three projecting α-helices running on the surface of the α/β core.

Two subunits form a very tight homodimer with a buried interface of 3,027 Å^2^, bringing the two zinc binding pockets close to each other. The juxtaposition of the two β2 strands at an angle of 120° results in the formation of an eight-strand β-sheet running through the core of the homodimer. Via domain swapping, the N-terminal arm of one molecule reaches both the zinc binding core and the C-terminal subdomain of the second molecule in the asymmetric unit, creating a broad and deep groove. The overall structure of the homodimer is reminiscent of a saddle (Figure 2A).

### The zinc binding site

**Figure 3:**
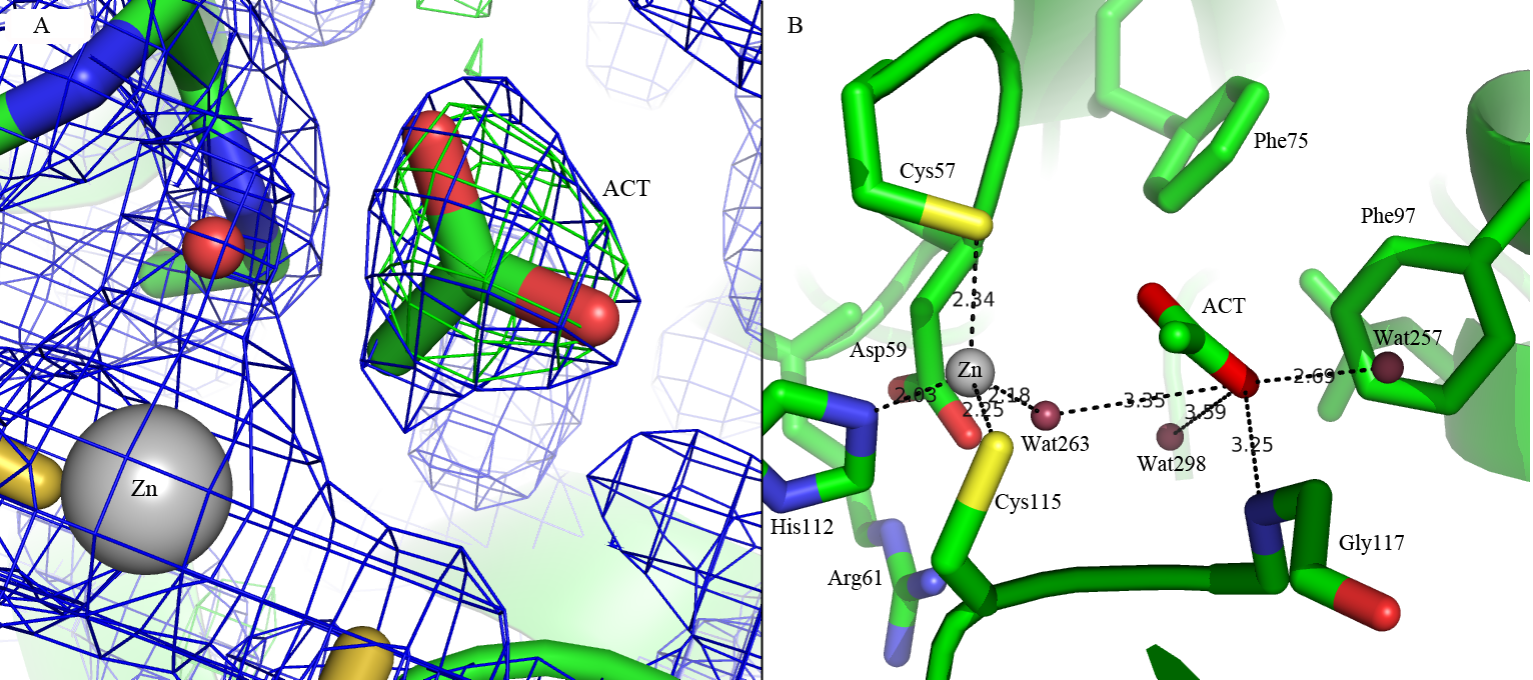
**The active site of Nce103**. **A) **Electron density map of the acetate molecule. The 2Fo-Fc map is colored in blue and contoured at 1.5 σ and the Fo-Fc map is colored in green and contoured at 3.0 σ. **B) **The coordinating bonds of the zinc ion and the hydrogen bonds stabilizing the acetate molecule are illustrated.

The active site is located at the inner side of the parallel β-sheet, thus largely sequestered from the solvent. Each subunit contains a zinc ion that resides at the interface between two subunits, with three coordinating residues (Cys57, Cys115 and His112) from the same subunit, and a water molecule Wat263A/Wat260B as the fourth to complete the tetrahedral coordination (Figure 3B). The average coordination bonds of the two zinc ions are 2.29 ± 0.05, 2.05 ± 0.01, 2.32 ± 0.07 and 2.12 ± 0.05 Å for Cys57Sγ, His112Nε, Cys115Sγ and Wat263A/Wat260B, respectively. The four zinc ligands are stabilized by the surrounding residues via a number of hydrogen bonds: His112Nγ~Thr113O (carbonyl oxygen); His112Nε~Wat267O; Cys115Sγ~Gly117N and Cys57Sγ~Asp59N. In addition, two conserved residues Asp59 and Arg61 form two hydrogen bonds: Asp59Oδ1~Arg61NH1 and Asp59Oδ2~Arg61NH2.

### An acetate molecule in the substrate binding pocket

A hydrophilic tunnel that passes a bottleneck consisting of Phe97, Leu102, Gly111 and Asp59 is the only access to the active site of Nce103. In the substrate binding pocket, there is a flat trilobed piece of density, reminiscent of an acetate molecule (Figure 3A), which is an analog of the substrate bicarbonate as reported previously [11]. In both active sites of the two subunits, the acetate molecule is finely complementary to the pocket from both the viewpoints of shape and charge (Figure 3B). Moreover, the acetate is stabilized by five hydrogen bonds with the amide nitrogens of residue Gly117 and three water molecules (Figure 3B). On the surface of the homodimer, there are four flat but slightly longer pieces of electron density which are well fitted with four molecules of ethylene glycol. Both acetate and ethylene glycol were introduced during crystallization.

### Comparison between the active sites of Nce103 and β-CAs from other species

**Figure 4:**
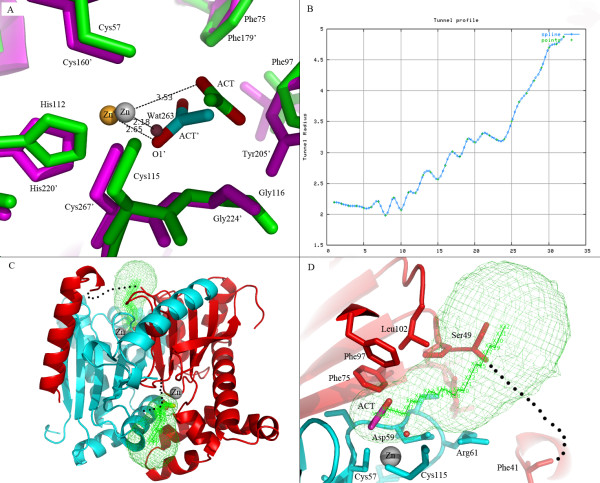
**The substrate tunnel of Nce103**. **A) **The superposition between the active sites of Nce103 and *P. sativum *β-CA (subunit A). Nce103 is shown in green and *P. sativum *β-CA in magenta. Zinc ions in Nce103 and *P. sativum *β-CA are colored in gray and orange, respectively. The water molecule Wat263 is colored in red. Residues and the acetate in *P. sativum *β-CA are marked with prime. **B) **The line graphs of the tunnel profile. The X-coordinate shows the path step; and the Y-coordinate shows the tunnel radius. **C) **The simulated tunnel of Nce103. Subunit A and B are colored in cyan and red, respectively. The tunnel is colored in green. The missing loop region is labeled as a dashed line. **D) **A closer look at the tunnel.

The crystal structure of Nce103 was superimposed on three β-CA homologs from *P. sativum *(PSCA; PDB code: 1ekj), *E. coli *(ECCA; PDB code: 2esf) and *C. neoformans *(Can2; PDB code: 2w3n), giving a root mean squared deviation (RMSD) of 1.58 Å, 1.96 Å and 1.95 Å, respectively, for 197 Cα-atoms. Moreover, the zinc-coordinating residues (Cys57, His112, Cys115) of Nce103 and those of PSCA (Cys160', His220', Cys267') superimpose well (Figure 4A). Besides in Nce103, an acetate molecule was also found in the active sites of PSCA and Can2, respectively. Compared to that in PSCA (or Can2), the acetate molecule (ACT) in Nce103 is far away from the zinc ion (3.53 vs 2.65 Å). The fourth zinc ligand Wat263 (Wat260 in subunit B) of Nce103 occupies the position of ACT'-O1' in PSCA, putting ACT away from the zinc ion (Figure 4A). The distance between the acetate molecule and the zinc ion in Nce103 (3.53 Å & 3.99 Å) and that in subunit A in PSCA (2.65 Å) are different, reminding us of the moving path of the substrate/product. In addition, a bicarbonate molecule was found in the noncatalytic binding site of ECCA, which is at the opposite of the zinc ion [5]. As another notable difference, the density map of the residues Pro41 to Gln48 in Nce103 is absent (Figure 2B), whereas the counterparts in most β-CAs of known structure are well ordered. However, superposition of these structures indicated that the loop adopts different conformations.

### A proposed tunnel of substrate

Using the program CAVER, we added hydrogen atoms to the Nce103 structure and calculated a solvent accessible pathway starting from the acetate ion to the outside bulky solvent. The tunnel profile is a graph of cross section radius (radius of maximally inscribed ball) versus tunnel length measured from its deepest place to the outside (Figure 4B). We speculate that this calculated pathway can mimic the possible substrate diffusion tunnel [21]. The two tunnels in the homodimer point to the opposite directions towards the surface (Figure 4C). It is worth noting that this tunnel is funnel-shaped, widely open toward the solvent but has a narrow gorge (cross section radius ~2.1 Å) in the vicinity of the acetate ion, suggesting that the linear CO_2 _(with a length of ~3.3 Å) molecule can freely diffuse toward the active sites, consistent with the diffusion-control catalytic mechanism of β-CAs. The gorge of the funnel is lined by several hydrophobic residues (Phe75, Phe97 and Leu102) on one side and polar residues (Asp59 and Arg61) on the other (Figure 4D). The distribution of hydrophobic and polar residues creates an ideal microenvironment for the diffusion of both nonpolar CO_2 _and negatively charged HCO3^-^. Furthermore, all these residues are highly conserved among β-CAs from other species, suggesting a potential role of these residues in assisting the substrate approaching the active site (Figure 1).

### Activity assays indicated that the N-terminal arm of Nce103 is indispensable

In the pathogenic fungus *Cryptococcus neoformans*, the N-terminal domain of the β-CA (Can2) is formed by four antiparallel α-helices [15]. It is reported that the N-terminal extension has a strong interaction with a surface groove which is located on the top of the active site of an adjacent monomer within the dimer. So, the N-terminal extension might mediate a regulation mechanism or protein/protein interaction [15]. The shorter N-terminal extension of Nce103 does not contain those residues necessary to interact with the active site in Can2 (Figure 1). To further validate the putative contribution of the N-terminal arm to the activity of Nce103, we constructed another truncated version of Nce103 which contains residues His51-Lys221, designated as Nce103ΔN50. During purification, both Nce103ΔN13 and Nce103ΔN50 were not stable at pH 8.5 (data not shown), despite Nce103ΔN50 remained dimeric in solution. Therefore, the Nce103 homodimer is mainly sustained by the interactions of the hydrophobic groups of the zinc binding core, instead of the swapped N-terminal arms.

**Figure 5:**
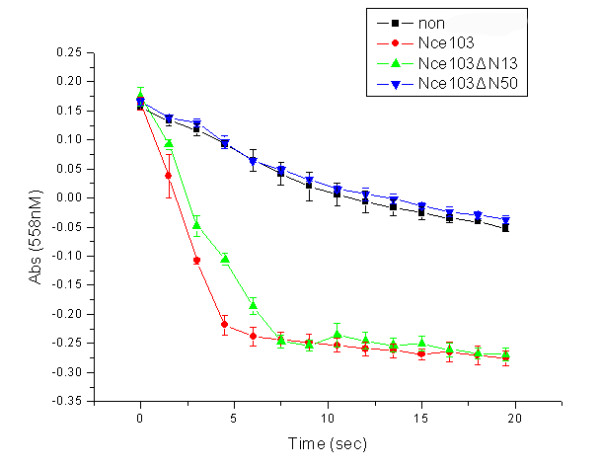
**Activity assays of Nce103 and its truncation versions**.

The activity of Nce103, Nce103ΔN13 or Nce103ΔN50 to catalyze the hydration of CO_2 _at pH 7.5 was determined using a colorimetric assay [22]. Nce103ΔN13 shows almost the same activity as the full length Nce103, whereas the activity of Nce103ΔN50 has been completely abolished (Figure 5). These results indicate that the first 50 residues are indispensable for the stability and activity of Nce103.

## Conclusion

Carbonic anhydrase is an important enzyme in inorganic carbon utilization. Here we provide the X-ray structure of *S. cerevisiae *β-carbonic anhydrase Nce103 at 2.04 Å resolution. The quaternary structure of Nce103 resembles the typical β-CAs of known structure. Comparative structure analysis enabled us to find a possible moving path of the substrate analog acetate toward the active site, and to calculate a funnel-shaped tunnel for the diffusion of the substrate/product. Furthermore, the activity assays indicated the N-terminal arm is indispensable for the enzymatic activity.

## Methods

### Cloning, expression and purification

The open reading frame of the *NCE103/YNL036W *gene was amplified by PCR using the genomic DNA of *S. cerevisiae *strain S288C as the template. An additional sequence coding for a six-histidine tag was introduced at the 5' end of the gene during PCR amplification. Then the PCR product was cloned into a pET29a-derived vector between *Nco *I and *Not *I restriction sites. Expression was done at 37°C using the transformed *E. coli Rossetta *(DE3) strain and 2× YT medium (OXOID LTD.) supplemented with 30 μg/ml kanamycin and chloramphenicol, respectively. When the cell culture reached an OD_600 nm _of 0.6, protein expression was induced with 0.2 mM IPTG (BBI) and the cells were grown for a further 4 hr. Cells were collected by centrifugation, resuspended in 30 ml buffer containing 200 mM NaCl, 20 mM Tris-HCl, pH 8.5. Cells were lysed by three cycles of freezing/thawing followed sonication. His-tagged proteins were purified using a Ni^2+ ^affinity column. Eluted protein was further purified by gel filtration using a Superdex™ 75 column (GE Healthcare Bioscience) equilibrated in 200 mM NaCl, 20 mM β-mercaptoethanol and 20 mM Tris-HCl, pH 8.5. The purity of the pooled fractions was checked by SDS-PAGE.

The truncated *NCE103 *without the sequence coding for the N-terminal 13 or 50 residues (Nce103ΔN13 and Nce103ΔN50) were amplified, respectively. PCR products were purified using the DNA gel extraction kit (V-gene, China) and inserted into pET29a-derived vector. The mutant proteins (Nce103ΔN13 and Nce103ΔN50) were overexpressed and purified as described above, except that Nce103ΔN13 for crystallization was purified in 20 mM acetate buffer, pH 4.6.

### Crystallization of Nce103ΔN13

Crystals of Nce103ΔN13 were obtained by the hanging drop vapor diffusion method at 16°C. In each drop of crystallization, 2 μl protein sample at 4 mg/ml in the buffer of 40 mM NaCl, 20 mM sodium acetate, pH 4.6 and 20 mM β-mercaptoethanol was mixed with 1 μl reservoir solution (20% PEG4000, 0.1 M sodium citrate, pH 5.6, 0.1 M sodium acetate, 20% ethylene glycol) and equilibrated against 0.5 ml reservoir solution. Crystals at a maximal size of 100-200 μm appeared within 6 days.

### Data collection and structure determination

The crystal was flash frozen at 100 K in a stream of nitrogen gas. In total 102 images of diffraction were collected using a MAR345dtb detector (MarResearch, Germany), with wavelength of 1.5418 Å and oscillation of 1 degree. X-ray crystallographic data were processed using the program *MOSFLM*. The structure was determined by molecular replacement method with the program *PHASER *[23] using the structure of β-CA from the red alga *Porphyridium purpureum *(PDB code: 1DDZ) as the search model. The initial model was refined by using the maximum likelihood method implemented in *REFMAC5 *as part of *CCP4i *program suite and rebuilt interactively by using the σ_A_-weighted electron density maps with coefficients 2mFo-Fc and mFo-Fc in the program *COOT *[24]. The final model was validated with the programs *PROCHECK *and *MOLPROBITY *and one monomer consists of residues Asn17-Phe40 and Ser49-Lys221 that fit well in the electron density map and 152 water molecules. Structure factors and the coordinates have been deposited in the Protein Data Bank (PDB) under the accession code of [PDB: 3EYX]. The final statistics and refinement parameters are listed in Table 1. The substrate accessing tunnel of Nce103 was calculated using the online version of *CAVER *[21] and all the structure figures were prepared using the program *PyMol *.

**Table 1:** Crystal parameters, data collection and structure refinement statistics.

***Data processing***	
Space group	*C*222_1_
Unit cell (Å), (°)	A = 60.57, b = 155.73, c = 89.69 α = β = γ = 90.00
Resolution range (Å)	16.11-2.04 (2.14-2.04)^a^
Unique reflections	25,657 (3,208)
Completeness (%)	93.1 (81.0)
<*I*/*σ (I)*>	13.5 (3.2)
R_merge_^b ^(%)	6.6 (31.0)
Average redundancy	2.8 (2.8)
*Refinement statistics*	
Resolution range (Å)	16.11-2.04
R-factor^c^/R-free^d ^(%)	19.70/24.14
Number of protein atoms	3,130
Number of water atoms	152
RMSD^e ^bond lengths (Å)	0.010
RMSD bond angles (°)	1.107
Mean B factors (Å^2^)	21.70
Ramachandran plot^f^(residues,%)	
Most favored (%)	97.41
Additional allowed (%)	2.59
Outliers (%)	0
PDB entry	3EYX

^a^The values in parentheses refer to statistics in the highest bin.

^b^R_merge _= ∑_hkl_∑_i_| I_i_(hkl)- <I(hkl)>|/∑_hkl_∑_i_I_i_(hkl), where I_i_(hkl) is the intensity of an observation and <I(hkl)> is the mean value for its unique reflection; Summations are over all reflections.

^c^R-factor =∑_h_| Fo(h)€-Fc(h)|/∑_h_Fo(h), where Fo and Fc are the observed and calculated structure-factor amplitudes, respectively.

^d^R-free was calculated with 5% of the data excluded from the refinement.

^e^Root-mean square-deviation from ideal values.

^f^Categories were defined by Molprobity.

### Enzymatic activity measurements

The assay was performed at 25°C and repeated twice by the changing pH/dye indicator method at pH 7.5 [22]. The 2× buffer indicator solution and enzyme of 100 μl contained (final concentrations): 25 mM HEPES, 100 μM phenol red (pH 7.5, λ max = 558 nm), 100 mM sodium sulfate and 2 μM enzyme. The reaction was initiated by addition of 100 μl CO_2 _(aq) to 100 μl of a 2× buffer indicator solution containing enzyme. The activity of Nce103 was measured at pH 7.5. A negative control was included in each set of measurements. The absorption at 558 nm was monitored for 20 sec at an interval of 1.5 sec using Beckman-DU 800 (Beckman Coulter LTD.).

## Authors' contributions

YBT cloned, expressed, purified and crystallized the protein Nce103, and performed the activity assays. YLJ, YXH and YBT performed structure determination, refinement and structure function analysis. WWH cloned the plasmid of full length Nce103. FML purified Nce103ΔN50. CZZ coordinated all the components of the project, and provided financial support. YBT and CZZ wrote the paper. All authors have read and approved the final manuscript.
